# Rumbles and Grumbles from Around the World

**DOI:** 10.1100/tsw.2000.19

**Published:** 2000-12-15

## Abstract

A different kind of shake-up will hit the science establishment when the New Year dawns in earthquake-prone Japan, reports Nature in its lead story this week. Science kicks off self-referentially with a lead story about its decision to publish a paper on sequencing the human genome from Craig Venter of Celera Genomics.

The latest skirmish in the protracted battle between the private gene-sequencing company led by Venter and the public Human Genome Project (HGP) has been raging behind the scenes for weeks, but it burst into the public eye last week. Eliot Marshall, a Senior Correspondent on the News Staff reports in *Science's* lead story (*Nature* put the story in slot 3) that on December 6, geneticist Michael Ashburner of Cambridge University e-mailed an open letter to reviewing editors at *Science* and members of the press. He slammed a decision by *Science* to allow Celera to keep control of the sequence by distributing it through its own Web site, reports the news staff of *Science*. Small pieces of the sequence will be available free to non-profit researchers, but Celera reserved the right to "place conditions," presumably profitable ones, on larger data requests and commercial users. The decision is a departure for the journal portion of the publication, reports Marshall. The journal traditionally "suggests" that authors deposit published gene sequences in GenBank, a public repository whose data anyone can access and use without restrictions, and altruistic scientists invariably do.

Donald Kennedy, Editor-in-Chief of *Science*, defended his decision with an appeal to enlightened capitalism. Marshall writes that Kennedy believes Celera has right to profit from its work, and requiring public release of the data would be an unreasonable precondition that would be tantamount to rejecting the paper outright. But many scientists privately believe the paper should be rejected, reports Marshall. Ashburner said he was "outraged and angry" that *Science* was not forcing Celera to deposit its sequence data in GenBank.

Geneticists are concerned that a troubling precedent has been set, report both magazines. "What will happen if someone else from the academic sector says, 'I have an interesting result to report, but I can't give you all the data'?" asks Harold Varmus, president of the Memorial Sloan-Kettering Cancer Center and former National Institutes of Health director. Another precedent may also have been set. An anonymous geneticist told Marshall that HGP has submitted its papers on the human genome to *Nature*. The source did not say whether or not this was a direct response to the decision by *Science*, but the HGP decision was made one day after Ashburner's letter went public, reports Marshall.

Controversy also figures in *Nature's* lead story. *Nature* reports that two new official bodies will come into operation when the Japanese bureaucracy reorganizes on January 6, 2001. The new bodies are the Council for Science and Technology Policy (CSTP) and the Ministry of Education, Culture, Sports, Science and Technology. Following the bureaucratic shuffle, the government will also shake up the national laboratories in April. The stated goal is to improve scientific research by removing a layer of bureaucracy, says *Nature*, but there are concerns that scandal may already have doomed one member of the new organization, CSTP minister Takashi Sasagawa. Sasagawa, a wealthy legislator, has recently been implicated in a tax evasion case that could cripple his ability to lead the CSTP. Despite the current troubles, *Nature* reports that scientists are generally happy with the re-organization and expect it to improve Japanese research in the long term. *Science* did not cover this story.
